# Visible Light Photo-Fenton with Hybrid Activated Carbon and Metal Ferrites for Efficient Treatment of Methyl Orange (Azo Dye)

**DOI:** 10.3390/molecules30081770

**Published:** 2025-04-15

**Authors:** Malak Hamieh, Nabil Tabaja, Khaled Chawraba, Zeinab Hamie, Mohammad Hammoud, Sami Tlais, Tayssir Hamieh, Joumana Toufaily

**Affiliations:** 1 Laboratory of Applied Studies for Sustainable Development and Renewable Energy (LEADDER), Doctoral School for Science and Technology (EDST), Lebanese University, Hadath P.O. Box 6573/14, Lebanon; malak_hamieh97@outlook.com (M.H.); nabiltabaja1@gmail.com (N.T.); khaled.chawraba@outlook.com (K.C.); zaynabahdhamei@gmail.com (Z.H.); joumana.toufaily@ul.edu.lb (J.T.); 2 Laboratory of Materials, Catalysis, Environment and Analytical Methods (MCEMA), Faculty of Science, Lebanese University, Hadath P.O. Box 6573/14, Lebanon; 3 Chemistry Department, Faculty of Sciences, ALAYEN Iraqi University (AUIQ), Alayen 8530, Iraq; 4 TIMR (Integrated Transformations of Renewable Matter), Royallieu Research Center, University of Technology de Compiegne, ESCOM, CS 60 319, CEDEX, 60-203 Compiegne, France; 5 Bahaa and Walid Bassatne Department of Chemical Engineering and Advanced Energy, Faulty of Engineering and Architecture, American University of Beirut, Beirut P.O. Box 11-0236, Lebanon; msh47@mail.aub.edu; 6 College of Engineering and Technology, American University of the Middle East, Egaila 54200, Kuwait; sami.tlais@aum.edu.kw; 7 Faculty of Science and Engineering, Maastricht University, P.O. Box 616, 6200 MD Maastricht, The Netherlands

**Keywords:** water purification, photocatalysis, metal ferrites, nanoparticles, SBA-15, methyl orange

## Abstract

Ensuring effective water purification is essential for addressing freshwater scarcity and achieving the United Nations Sustainable Development Goals (SDGs). An efficient hybrid mixture, composed of FeCr quantum dots doped into mesoporous silica SBA-15 support and activated carbon (AC) derived from olive mill solid wastes, has been developed for treating high optical density polluted aqueous environments. This hybrid, denoted as FeCr-SBA-15/AC, was examined for its efficacy in the adsorption and photo-Fenton degradation of met orange dye (MO), a model high-optical-density pollutant, under visible light exposure. Characterization of the prepared samples was conducted using X-ray diffraction (XRD), Fourier transform infrared spectroscopy (FT-IR), Brunauer–Emmett–Teller (BET) surface area analysis, diffuse reflectance spectroscopy (DRS), scanning electron microscopy (SEM), and transmission electron microscopy (TEM). Key parameters investigated included catalyst dosage, dye concentration, solution pH, and H_2_O_2_ concentration. Remarkably, the FeCr-SBA-15/AC hybrid exhibited superior photocatalytic activity, achieving a degradation efficiency of 97% for MO under optimized conditions (catalyst dosage = 0.75 g L^−1^, dye concentration = 20 mg L^−1^, pH = 5.47, and 0.5 mL H_2_O_2_) after 180 min of irradiation with visible light. This performance surpassed that of FeCr-SBA-15 alone by 20%, due to the synergistic effects of adsorption and photo-Fenton. The adsorption of MO onto AC followed the Freundlich model equilibrium isotherm, while the experimental data for the hybrid mixture aligned well with the pseudo-first-order Langmuir–Hinshelwood kinetic model with a rate constant of 0.0173 min^−1^. The leaching of Cr in the solution was very low—0.1 ppm—which is below the detection limit. These findings underscore the potential of the synthesized FeCr-SBA-15/AC hybrid as a cost-effective, environmentally friendly, and highly efficient photo-Fenton catalyst for treating wastewater contaminated by industrial effluents.

## 1. Introduction

The substantial growth in population and industrial expansion has exerted significant pressure on available water resources, resulting in a notable scarcity of safe drinking water globally [1]. The reports revealed that around 1 billion people lack access to clean and safe water, contributing to 2 million deaths annually [2]. Notably, dye effluents from textile industries are considered one of the major sources of water pollution [3]. Approximately 100,000 different types of dyes are commercially available worldwide, with azo dyes constituting the largest category (70%) [4]. Around 1 × 10^6^ tons are produced every year, and 15% of them are discharged into water streams during the dying process [5]. These dyes have complex aromatic molecular structures which make them very stable and difficult to biodegrade [6]. Even in small amounts, they can be highly toxic.

Various technical methods are employed for wastewater purification, including adsorption [7,8], coagulation-flocculation [9,10], ion exchange [11], bioremediation [12], filtration [13], and advanced oxidation processes [14,15]. Advanced oxidation processes relying on heterogeneous photo-Fenton present notable efficacy in eliminating micropollutants found in wastewater, even at extremely low concentrations. However, their applicability encounters limitations in industrial wastewater contexts, particularly those stemming from dye-utilizing industries. This is primarily due to both elevated dye concentrations and heightened optical density, even at relatively low dye concentrations (>20 ppm). The high concentration of contaminants in wastewater leads to the saturation of active sites on the catalyst surface, which reduces light penetration and subsequently decreases hydroxyl radical formation. AC is a versatile material widely used as adsorbent in wastewater treatment applications due to its large surface area and well-developed porous structure [16]. The high AC adsorption capacity will reduce the concentration of dyes in the solution before photodegradation, thereby increasing light penetration into the semiconductor surface and promoting the production of •OH. However, despite its numerous advantages, AC cannot be used alone as a primary treatment method, because it only concentrates the pollutants without further degradation. Therefore, there is a need for a secondary treatment method to remove the adsorbed pollutants and regenerate the catalyst. AC can combine with various semiconductors to form composite photocatalysts [17,18,19]. The synergistic effect of the AC and photocatalyst mixture can be attributed to the formation of an interface between the two solids. In this arrangement, the AC adsorbs pollutants near the catalyst, facilitating their transport via surface diffusion to the photocatalytic sites for decomposition [20]. In their study, Matos and coworkers showed that the addition of AC into TiO_2_ under UV light has a beneficial effect on the photodegradation of methyl orange (MO), phenol, and herbicides [21]. Kadirova et al. [22] studied the adsorption and photodegradation of methylene blue by iron oxide impregnated on AC. The impregnated sample (AC-Fe_2_O_3_) provides an effective composite for the degradation of MB. Thus, by combining the high catalytic performance of the photocatalyst with the affordability, reusability, and excellent adsorption capacity of AC, this composite is expected to offer an economical and effective method for the photodegradation of MO.

TiO_2_ and ZnO are widely employed catalysts in wastewater remediation purposes; however, their wide band gap limits their ability to effectively utilize solar energy, as they can only absorb photons with wavelengths shorter than 390 nm [23]. Because of that, scientists are searching for catalysts that can absorb light in the visible region of spectrum. Recently, spinel-type metal ferrite nanoparticles (MFe_2_O_4_) have attracted considerable interest as heterogeneous photo-Fenton catalysts due to their low cost, high chemical stability, small particle size and excellent magnetism [24,25]. Numerous studies have demonstrated that MFe_2_O_4_ catalysts exhibit strong photodegradation capacity, making them highly effective for the complete removal of contaminants [26]. Because of their magnetic properties, MFe_2_O_4_ nanoparticles can be conveniently removed from the mixture using an external magnetic field. Additionally, MFe_2_O_4_ semiconductor exhibits a narrower band gap (2.2 eV) [27,28], which enhances their photo-Fenton performance under visible-light absorption irradiations. To enhance the dispersion of MFe_2_O_4_, increase the specific surface area, reduce leaching, and prevent the recombination of photogenerated electron-hole pairs, MFe_2_O_4_ is typically loaded onto various supports such as zeolites, silica, and clay. Reports have revealed that the dispersion of metallic nanoparticles into porous materials increases their catalytic performance towards the degradation of contaminants by the heterogeneous photocatalysis method. Santa Barbara Amorphous (SBA) materials are mesoporous silica known for their uniform pore sizes (ranging from 4.6 to 30 nm), well-defined pore structure and size distribution, high surface area, excellent thermal stability [29], and ability to support a variety of active species. The literature describes several types of SBA materials, such as SBA-1 (Pm3n, cubic), SBA-15 (P6mm, hexagonal), and SBA-16 (Im3m, cubic). Among these, SBA-15 stands out due to its numerous applications, including selective adsorption, heterogeneous catalysis, various chemical transformations, and gas storage [30].

The advanced oxidation process (AOP), utilizing SBA-15 mesoporous silica and hydrogen peroxide (H_2_O_2_), has shown significant promise in water decontamination, particularly for the degradation of various organic contaminants. SBA-15, due to its unique physico-chemical properties, such as high specific surface area, uniform hexagonal pores, and chemical inertness, serves as an excellent support material for catalytic elements. These elements, like iron (Fe) and copper (Cu), enhance the generation of hydroxyl radicals (•OH) when interacting with H_2_O_2_, which are crucial for breaking down pollutants. In heterogeneous Fenton-like processes, SBA-15-based catalysts, such as Fe-SBA-15 or Cu-SBA-15, activate H_2_O_2_ to produce •OH radicals, which attack and decompose the organic contaminants into less harmful compounds like CO_2_ and H_2_O. The incorporation of catalytic elements into SBA-15 not only improves the dispersion of the active sites, but also prevents the leaching of metal ions, thus enhancing the stability and reusability of the catalyst. Studies have demonstrated the superior performance of bimetallic SBA-15 composites (e.g., Cu-Fe-SBA-15) over their monometallic counterparts in the degradation of complex organic molecules, achieving high removal efficiencies within relatively short reaction times. Additionally, the confinement effect of the mesoporous structure enhances the local density of active sites, thereby improving the overall catalytic activity. Mechanistic studies reveal that the increased efficiency of these SBA-15-based catalysts is due to the improved generation and utilization of reactive radicals. Furthermore, factors such as reaction temperature, pH, and H_2_O_2_ concentration significantly influence the oxidation rate and efficiency, with higher temperatures and optimal pH levels enhancing the degradation process [29,30,31].

In the current work, we aim to synthesize a mixture of oxide nanoparticles using co-dopant metals Fe and Cr, dispersed on SBA-15 silica support. Herein, FeCr-SBA-15 was combined with AC from olive mill solid wastes (OMSW). The objective was to develop a hybrid mixture made of two components—an adsorbent capable of adsorbing the contaminants and a photo-Fenton catalyst responsible for their mineralization. The hybrid mixture, FeCr-SBA-15/AC, was utilized to study the synergic effect of adsorption and photo-Fenton in the degradation of MO as a model dye under visible light. MO, a common anionic azo dye, is difficult to degrade due to the stability of its aromatic structures against light and oxidation [32]. As a primary pollutant in wastewater, MO poses severe risks to human health, aquatic ecosystems, and the environment due to its toxicity, non-biodegradability, and carcinogenicity [6]. The treatment for the degradation of MO dye in water is of utmost importance due to its widespread presence in industrial waste, posing a significant threat to the environment and human health. The degradation of methyl orange dye has also been studied in the context of oxidative systems, such as persulfate and quasi-monochromatic UV radiation treatment [33]. Therefore, it is crucial to treat wastewater containing MO and develop efficient treatment methods to remove it before it is discharged into the environment. The influence of different mass ratios between FeCr-SBA-15 and AC, pH, catalyst dosage, and MO concentration was discussed.

## 2. Results and Discussion

### 2.1. X-Ray Diffraction (XRD)

**Figure 1:**
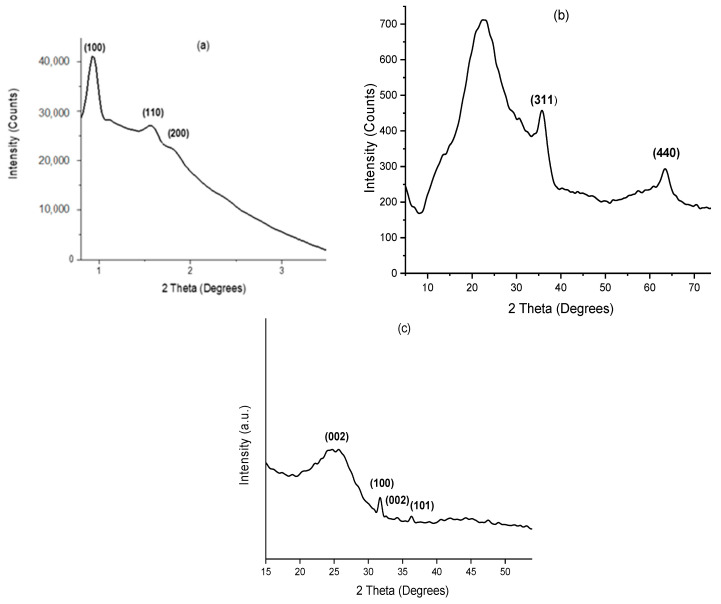
(**a**) SAXRD of SBA-15, (**b**) WAXRD of FeCr-SBA-15, and (**c**) WAXRD of AC.

The small-angle X-ray diffraction pattern (SAXRD) of the calcined SBA-15 is shown in Figure 1a. The SBA-15 sample exhibited three well-resolved diffraction peaks in the range 2*θ* = 0.4–2.0° that can be indexed to (100), (110), and (200) planes [34]. These peaks are associated with the two-dimensional p6mm hexagonal mesoporous structure typical for SBA-15. Figure 1b shows the wide-angle X-ray diffraction pattern (WAXRD) of FeCr-SBA-15 after calcination at 700 °C.

The diffractogram pattern reveals two diffraction peaks at 35° and 63° (2*θ*). The two primary peaks correspond to the (311) and (440) planes in a face-centered cubic lattice [35], indicating the presence of a spinel phase of CrFe_2_O_4_ (ICDD PDF 00-034-0425). This phase is comparable to the structures reported for magnetite (Fe_3_O_4_) or maghemite (γ-Fe_2_O_3_), both of which belong to the space group Fd-3m. The lack of an indexed diffraction peak at (111), typically observed at low angles, further supports the assignment to the maghemite (γ-Fe_2_O_3_) structure. Therefore, CrFe_2_O_4_ has an inverse spinel structure. In this instance, half of the trivalent Fe^3+^ and Cr^3+^ ions are evenly distributed between octahedral and tetrahedral sites, while the Fe^2+^ cations occupy the tetrahedral sites.

The XRD of AC (Figure 1c) showed a broad diffraction peak at around 23°, corresponding to the (002) plane of graphitic carbon materials. This broad peak indicated the prevalent amorphous structure of AC. Additionally, low intensity diffraction peaks were observed in the 32–36° range, which can be indexed to (100), (200), and (101) planes [36]. These peaks are consistent with the hexagonal wurtzite structure of ZnO (ICCD PDF 01-075-7070). The appearance of the ZnO peak indicates that residual ZnO particles remain on the activated carbon surface after the activation process, possibly due to incomplete removal during the washing step.

### 2.2. Nitrogen Adsorption–Desorption Isotherms

**Figure 2:**
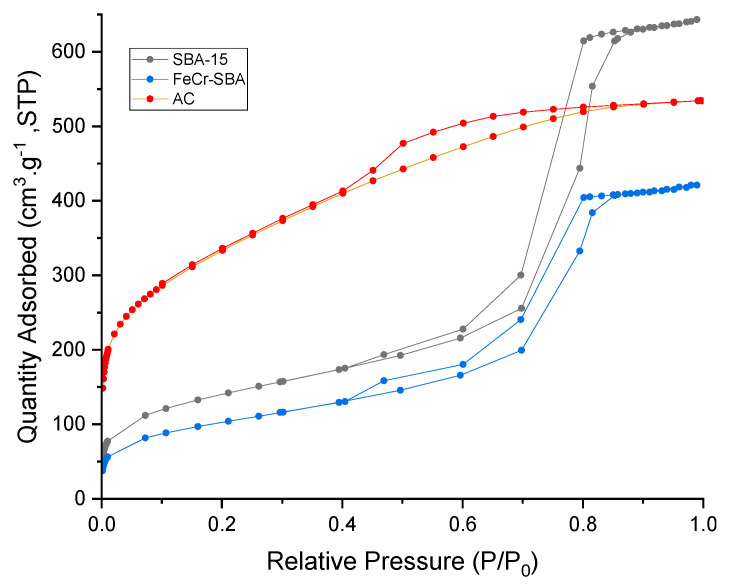
N_2_ adsorption–desorption isotherm of SBA-15, FeCr-SBA-15, and AC.

The textural properties of SBA-15, FeCr-SBA-15, and AC were analyzed using N_2_ adsorption–desorption isotherm. The results are displayed in Figure 2. The isotherms of SBA-15, and FeCr-SBA-15 are of type IV and exhibit a clear H1 hysteresis loop according to IUPAC classification, indicating mesoporous characteristics with a capillary condensation phenomenon [37]. The shape of the isotherm was preserved after incorporating Fe and Cr into SBA-15, indicating that the deposition of metals does not affect the textural properties of the support. The AC isotherm belongs to the category of type I, which is characteristic of microporous materials. Additionally, a hysteresis loop of type IV was revealed within the relative pressure range of 0.4 to 0.8. This loop indicates the existence of mesopores within the activated carbon structure. Thus, the prepared AC is a mixture of both micropores and mesopores.

The corresponding textural properties of all samples are summarized in Table 1. The BET surface area of SBA-15 was 522 m^2^ g^−1^, and its pore volume was 1.48 cm^3^ g^−1^. After the deposition of Fe and Cr metals onto the SBA-15 support, the surface area and pore volume of SBA-15 were reduced to 372 m^2^ g^−1^ and 0.65 cm^3^ g^−1^, respectively. The decrease in the surface area indicates that chromium ferrite nanoparticles are successfully incorporated into the mesopores of SBA-15. The BET surface area of AC is much higher (1148 m^2^ g^−1^) compared to those of SBA-15 (522 m^2^ g^−1^) and FeCr-SBA-15 (372 m^2^ g^−1^).

### 2.3. Fourier Transform Infrared (FT-IR)

**Figure 3:**
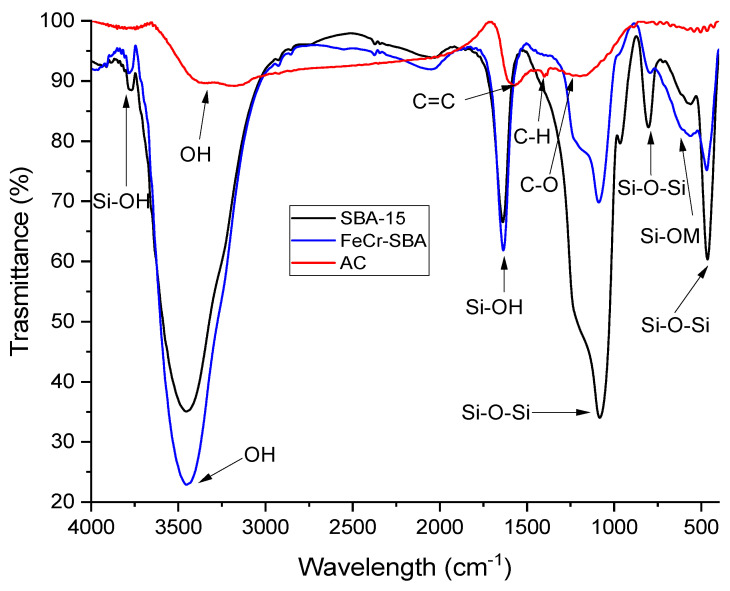
FT-IR spectra of SBA-15, FeCr/SBA-15, and AC.

The FT-IR profiles of SBA-15, FeCr/SBA-15, and AC are displayed in Figure 3. The SBA-15 sample exhibits absorption bands at 460, 803, and 1082 cm^−1^, characteristic of the stretching vibrations of the siloxane framework, Si-O-Si. An intense band was observed at 1630 cm^−1^, referring to the vibration of Si-OH bending mode [38]. The weak band at 970 cm^−1^ is ascribed to asymmetric stretching of Si-OH bond [39].

The small peak at 3762 cm^−1^ is associated with OH stretching and vibration modes of silanol groups inside the micropores of SBA-15 [40]. A broad absorption band at 3440 cm^−1^ indicates stretching vibrations of surface OH moieties. After functionalization, the spectrum of FeCr doped SBA-15 showed slight changes compared to the SBA-15 support, which could be attributed to the interaction of metal (Fe and Cr) with the surface of Si-OH, leading to the formation of metal–oxygen (O-M) interaction. The spectrum of FeCr doped SBA-15 showed a broad band at 560–600 cm^−1^, corresponding to the absorption of the transition metals Fe and Cr within their respective metal oxides, FeO and CrO. The FT-IR spectrum of AC showed a broad peak at 3350 cm^−1^, which refers to O-H stretching vibration. The peak at 1570 cm^−1^ can be associated with C=C stretching vibrational frequency of aromatic rings. Moreover, the band at 1385 cm^−1^ could be assigned to C-H bending mode [41]. The peaks at 1235 cm^−1^ are likely due to C-O stretching vibrations, which can be attributed to the C-O bonds found in alcohol, acid, phenol, ether, and/or ester functional groups.

### 2.4. Scanning Electron Microscope (SEM)–Energy Dispersive Spectroscopy (EDS)

**Figure 4:**
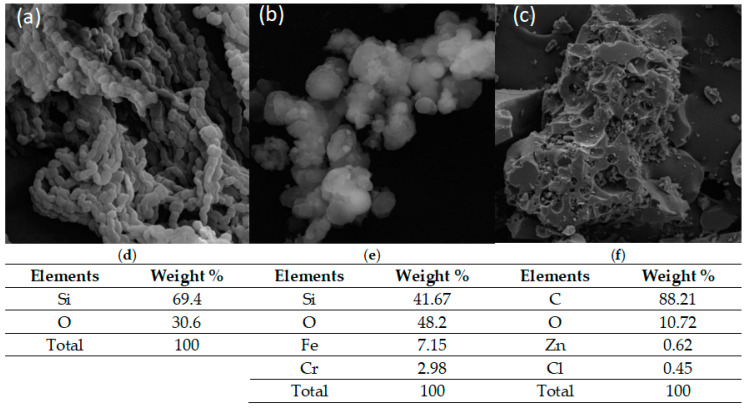
SEM micrographs and EDX results of (**a**,**d**) SBA-15 (**b**,**e**) FeCr-SBA-15, and (**c**,**f**) AC.

SEM equipped with EDS is used to study the morphology and elementary composition of the prepared samples. The images of SBA-15, FeCr-SBA-15, and AC are displayed in Figure 4. The SBA-15 grains revealed in Figure 4a are broad and substantial. However, the micrograph of FeCr-SBA showed spherical aggregates inside the pores of SBA-15. Although the deposition of chromium ferrite within the pores is not clearly observable through microscopy, it is anticipated that chromium ferrite nanoparticles are present inside the pores of SBA-15, based on the synthesis procedure used. Based on the process of Tabaja et al., the micrographs revealed the absence of chromium ferrite nanoparticles on the external surface of SBA-15; instead, these nanoparticles are formed within the pores of SBA-15 template when using iron nitrate precursors in the double solvent process. The image of AC shows a rough surface characterized by various cavities of different sizes and shapes. Moreover, scattered salt particles are present on the surface of AC, which originate from residual zinc salt.

The EDS results (Figure 4e) showed that FeCr-SBA-15 nanoparticles contain silicon (69.4%), oxygen (48.2%), iron (7.15%), and chromium (2.98%), compared to silica support SBA-15 which is composed of only two components—silicon (69.4%) and oxygen (48.2%) (Figure 4d). This confirms the successful incorporation of iron and chromium metals into the SBA-15 support. However, EDS analysis of AC (Figure 4f) showed carbon (88.21%) and oxygen (10.72%) with trace amounts of zinc (0.62%) and chlorine (0.45%).

### 2.5. Transmission Electron Microscope (TEM)

**Figure 5:**
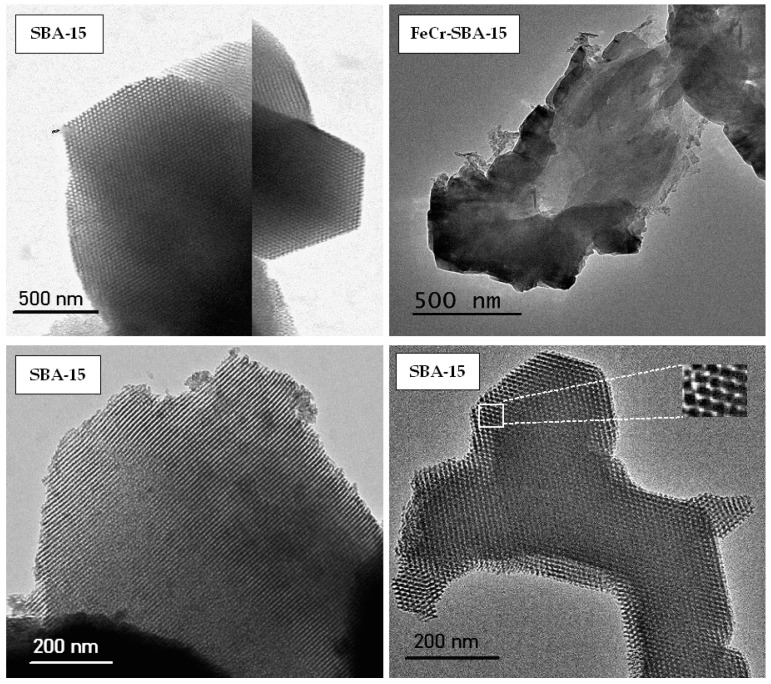
TEM images of SBA-15 and FeCr-SBA-15.

The morphologies of SBA-15 and doped FeCr-SBA-15 were investigated by TEM, and the results are displayed in Figure 5. The TEM image of SBA-15 clearly shows a highly ordered array of straight, parallel channels with hexagonally packed dark and bright contrasts, consistent with the long-range mesoscopic order characteristic of SBA-15 materials. This structural ordering is indicative of a 2D hexagonal p6mm symmetry, which is typical of SBA-15 viewed along the (100) direction. This interpretation was previously supported by a study of Zhao et al. [42], in which similar features were attributed to this symmetry.

For the FeCr-SBA-15 nanoparticles, the image exhibits aggregated, less ordered morphologies, with dense black regions corresponding to the incorporated FeCr oxide nanoparticles. The darker areas inside the mesopores indicate the presence of metal oxide domains, while the lighter grey regions represent the silica walls [43].

### 2.6. UV-Vis Spectroscopy

**Figure 6:**
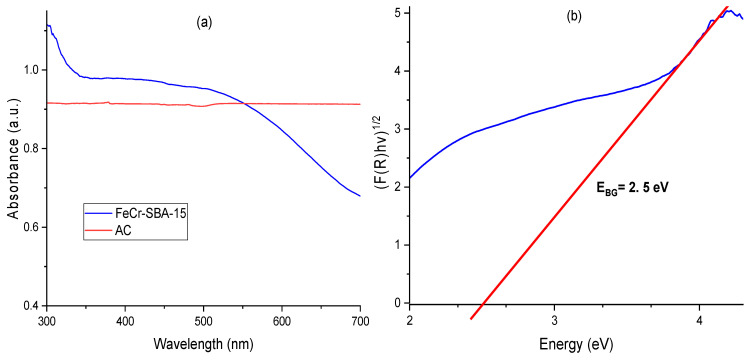
(**a**) UV-Vis diffuse reflectance spectrum of AC and FeCr-SBA-15 and (**b**) Tauc plot for FeCr-SBA-15.

The optical properties of semiconductors play a dominant role in their photo-Fenton activity. The UV-Vis diffuse reflectance spectrum of AC and FeCr-SBA-15 is depicted in Figure 6. From the results, it is evident that AC reveals no distinct absorption band in the visible region (400–700 nm). Similarly, SBA-15 does not show an absorption band in the range 300–700 nm [44]. However, the absorption results of FeCr-SBA-15 showed a broad absorption band within the visible spectrum range of 400–700 nm (Figure 6a). This shows that the prepared FeCr-SBA-15 catalysts act as effective photosensitizers, absorbing a considerable amount of visible light, enabling them to perform photocatalytic reactions under visible light irradiation. For the determination of the band gap (*E_BG_*), the Kubelka–Munk method and Tauc plots were applied [45]. FeCr-SBA-15 exhibited an indirect band gap (n = 2). The band gap was assessed by plotting (*F*(*R*)*hv*)^1/2^ as a function of the energy (*E*). The Tauc plot contains a part where a linear correlation exists. The extrapolation of the linear line onto the *x*-axis gives the value of the *E_BG_*, as demonstrated in Figure 6b. The *E_BG_* of FeCr-SBA-15 was 2.5 eV. An indirect band gap of 2.7 eV was reported in Ref. [46].

### 2.7. Adsorption of MO over Hybrid Mixture

**Figure 7:**
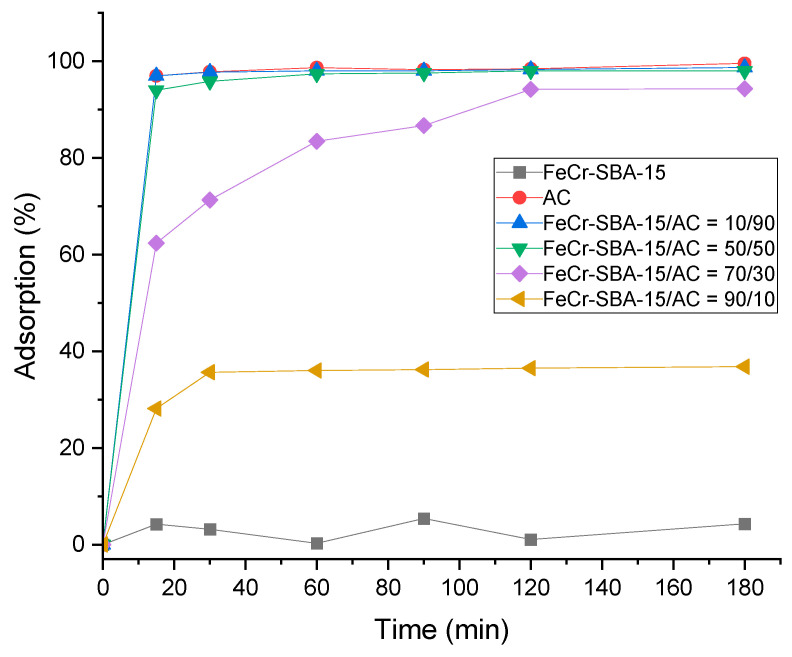
MO adsorption using AC, FeCr-SBA-15, and different ratios of FeCr-SBA-15/AC hybrid mixtures (20 mg L^−1^ MO, dose: 0.75 g L^−1^ and pH = 5.47).

Prior to testing the catalytic activity of the prepared hybrid mixture in the photocatalytic degradation of MO, the effect of the adsorption process was assessed using AC, FeCr-SBA-15, and different sets of hybrid mixture ratios of FeCr-SBA-15/AC (10/90, 50/50, 70/30, 90/10). The prepared samples were tested in the dark for 180 min, and the results of MO adsorption are shown in Figure 7. From the results, it can be seen that complete removal of MO (97%) was achieved using AC alone within 30 min. Additionally, high removal of MO was observed when using the following hybrid mixture ratios (10/90, 50/50, and 70/30), where AC dominates. This can be attributed to the high surface area of AC in the system, which promoted higher adsorption of MO. Meanwhile, FeCr-SBA-15 showed almost negligible removal of MO (4%) within 180 min. In our work, we aimed to study the photo-Fenton degradation of MO dye using a hybrid system of AC from OMSW and FeCr doped into SBA-15 silica support. The different mixtures (10/90, 50/50, and 70/30) were not used in the photo-Fenton degradation process because it is very difficult to attribute the high removal of MO to one of the two phenomena—adsorption or degradation. The hybrid mixture of 90/10 was chosen as the optimal ratio to be used in the further degradation experiments. This ratio showed a maximal removal percentage of 35% when the reaction was kept in the dark for 180 min.

### 2.8. Photo-Fenton Degradation of MO

**Figure 8:**
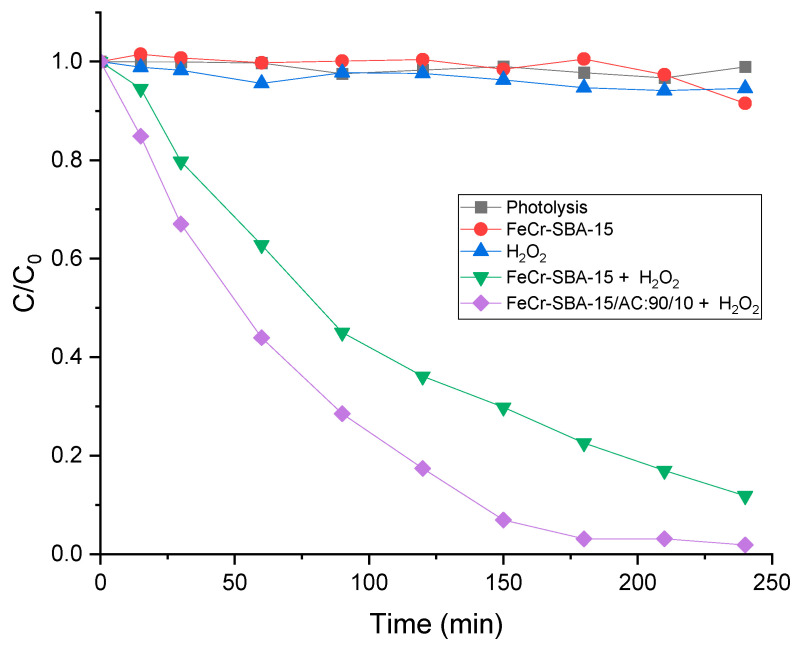
Degradation efficiency of MO under different experimental conditions (20 mg L^−1^ MO, 0.75 g L^−1^ catalyst, 0.5 mL H_2_O_2,_ and pH = 5.47).

For the photo-Fenton degradation of MO, several experiments were performed under visible light irradiation, and the results are shown in Figure 8. No significant MO degradation was observed during photolysis where we achieved only 1% degradation efficiency after 4 h of reaction, which indicates that the photolysis of MO was very weak and negligible. Additionally, it can be observed that there is almost no degradation of MO using FeCr-SBA-15 alone without H_2_O_2_ under visible light. Meanwhile, H_2_O_2_ alone accounts for only 5% degradation after 240 min. This is due to the limited oxidation ability of H_2_O_2,_ where only small amounts of •OH are produced from H_2_O_2_ during the reaction. In the presence of only 0.5 mL H_2_O_2_ (35%), FeCr-SBA-15 achieved 77% degradation of MO after 180 min. The degradation efficiency of MO was enhanced using the hybrid system of 90/10, resulting in a maximum degradation of 97% after 180 min. It is worth noting that the hybrid mixture (90/10) reached a maximum adsorption percentage of 35% within 180 min. Subsequently, the additional removal of 97% MO was primarily due to photodegradation itself. The AC in the hybrid mixture provides a synergetic effect on the photodegradation of MO. Its high surface area (1184 m^2^ g^−1^) allows for the efficient adsorption of the MO pollutant, facilitating the diffusion of MO from AC to the photoactive FeCr-SBA-15 surface. This results in high concentration of MO near FeCr-SBA-15 compared to the bulk solution, ultimately boosting the degradation of MO. In addition, AC acts as an electron trap, reducing electron-hole recombination and thereby improving the photo-Fenton activity of FeCr/SBA-15.

### 2.9. Influence of H_2_O_2_ Concentration

**Figure 9:**
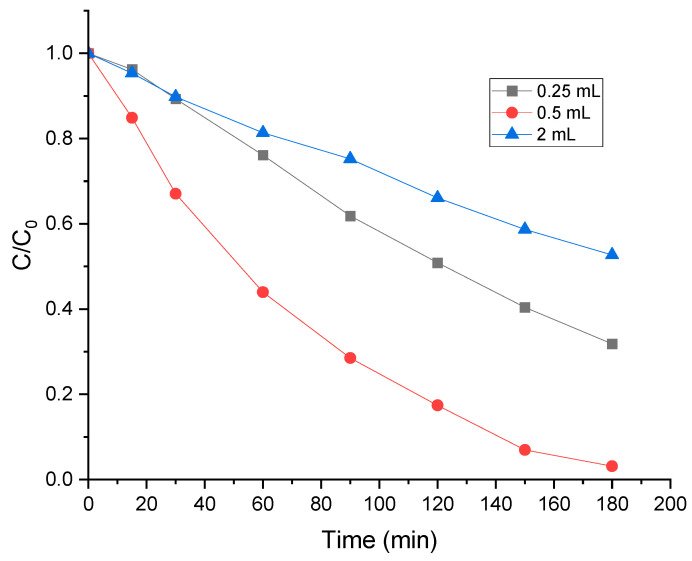
Effect of H_2_O_2_ concentration on MO degradation by hybrid mixture (20 mg L^−1^ MO, 0.75 g L^−1^ FeCr-SBA-15/AC, and pH = 5.47).

In photo-Fenton, H_2_O_2_ oxidant is considered a primary contributor to dye decomposition, as it enhances the generation of reactive oxygen species (ROS) and promotes radical reactions, thereby increasing dye degradation efficiency [47]. The effect of H_2_O_2_ concentration on the degradation of MO was assessed by changing the volume from 0.25 to 2 mL. As shown in Figure 9, the degradation efficiency rose from 49% to 64% in 120 min with an increase in the amount of H_2_O_2_ from 0.25 mL to 0.5 mL. The improvement in the degradation efficiency as the dosage of H_2_O_2_ increases is due to the production of more •OH radicals. However, when the amount of H_2_O_2_ exceeded 0.5 mL, the degradation efficiency of MO decreased. Thus, at high H_2_O_2_ concentrations, the excess H_2_O_2_ acts as a scavenger of •OH and forms hydroperoxyl radicals (Equation (1)) [48]. These radicals show reduced oxidation capacities (Equation (2)) which resulted in a lower degradation rate [49]. The optimal quantity of H_2_O_2_ for the photo-Fenton degradation of MO was 0.5 mL.

(1) $$H_{2}O_{2\;}+\cdot OH\;\to \;H_{2}O\;+\cdot OOH\;$$

(2) $$\cdot OOH+\cdot OH\;\to \;H_{2}O\;+O_{2}$$

### 2.10. Influence of Catalyst Dose on the Degradation of MO

**Figure 10:**
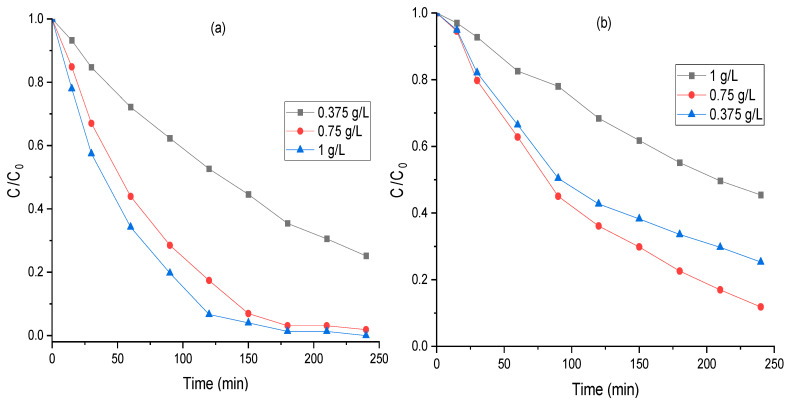
Effect of dose of (**a**) hybrid mixture and (**b**) FeCr-SBA-15 on the degradation efficiency of MO (20 mg L^−1^ MO, 0.5 mL H_2_O_2,_ and pH = 5.47).

Determining the optimal catalyst loading is critical in photodegradation reactions because it helps in scaling up the photocatalytic process and influences the overall economics of the process. The impact of FeCr-SBA-15 and the hybrid mixture dosage on MO degradation was studied by varying the quantity of catalyst from 0.0375 g L^−1^ to 1 g L^−1^ under similar experimental conditions. The results of MO degradation with time as a function of hybrid mixture loading are presented in Figure 10. The results demonstrate a clear enhancement in the degradation of MO from 47% to 93% along with an increase in the hybrid mixture dosage from 0.375 g L^−1^ to 1 g L^−1^. This result can be explained by the increase in the number of available active sites on the catalyst surface for the same unit volume of MO, which in turn increased the number of absorbed photons. Consequently, the production of hydroxyl radicals increased, resulting in a significant improvement in the degradation efficiency of MO. For FeCr-SBA-15, as the catalyst dosage increased from 0.375 g L^−1^ to 0.75 g L^−1^, there was a corresponding rise in the degradation of MO, from 31% to 64%. However, further increase in FeCr-SBA-15 dosage to 1 g L^−1^ lead to a slight decrease in MO, to 57%. This reduction in the degradation beyond the catalyst quantity of 1 g L^−1^ can be attributed to two main reasons. First, the high catalyst dose leads to an increase in the opacity and turbidity of the solution, which hinders the penetration of light. Second, the nanoparticles tend to agglomerate at high catalyst concentrations [50]. As a result, part of the catalyst surface may be unavailable for photon absorption and solute adsorption, consequently decreasing photocatalytic activity.

### 2.11. Influence of Initial MO Concentration

**Figure 11:**
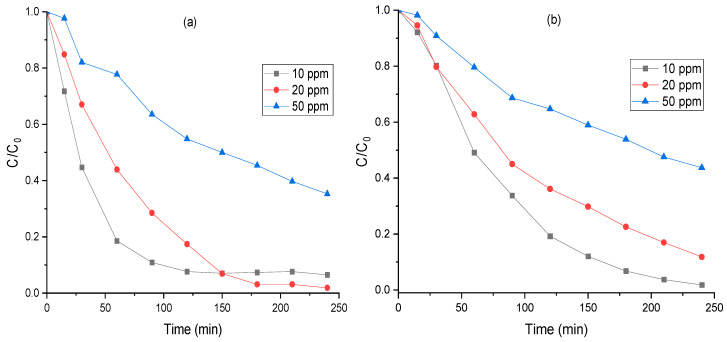
Effect of initial MO concentration on the degradation efficiency of MO using (**a**) hybrid mixture and (**b**) FeCr-SBA-15 (0.75 g L^−1^ catalyst, 0.5 mL H_2_O_2,_ and pH = 5.47).

The effect of initial dye concentration on the photocatalytic degradation of MO was investigated using FeCr-SBA-15 and hybrid mixture by varying the concentration from 10 ppm to 50 ppm under visible light irradiation. Figure 11 shows that the degradation efficiency reduced with increasing MO concentration. At a lower concentration of 10 ppm, around 92% of MO was degraded using the hybrid mixture within 120 min irradiation time. However, the degradation efficiency was reduced to 45% when the concentration was increased to 50 ppm. Similarly, for FeCr-SBA-15 catalyst, the degradation of MO decreased from 80% to 35% as the MO concentration increased from 10 ppm to 50 ppm. This decrease in MO degradation is likely attributed to the higher quantity of dye competing for degradation. Fewer photons can reach the hybrid mixture surface at very high concentrations because the solution screens out a large portion of light. As a result, the production of electron-hole pairs is reduced, which ultimately lowers the photodegradation performance.

### 2.12. Influence of Initial pH

**Figure 12:**
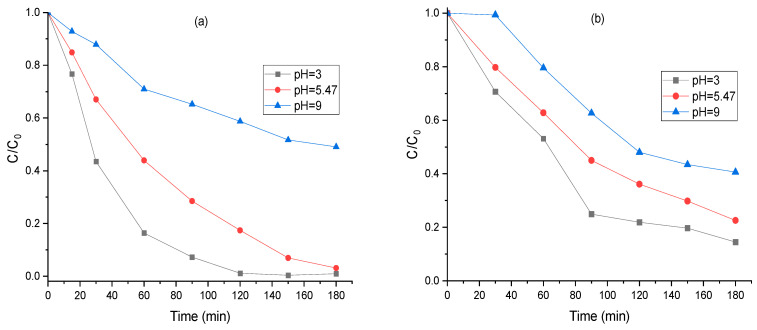
Effect of pH on the degradation efficiency of MO using (**a**) hybrid mixture (**b**) FeCr-SBA-15 (0.75 g L^−1^ FeCr-SBA-15/AC: 90/10, 20 mg L^−1^ MO, and 0.5 mL H_2_O_2_).

The pH is a critical parameter affecting the rate of degradation in the photocatalytic process. It has a significant effect on the charge distribution of the photocatalyst surface and influences the electrostatic interactions between the MO dye and the surface of photocatalyst in an aqueous solution. In addition, the pH of the solution influences the oxidation capacity of the formed radicals [51]. The color of MO shifts from yellow to orange and then to red as the solution becomes more acidic because of the hydrazone structure’s resonance [52]. The influence of solution pH on the rate of photocatalytic degradation of MO using the hybrid mixture and FeCr-SBA-15 was investigated within the range of 3–9 (Figure 12). The degradation efficiency of MO using the hybrid mixture decreased significantly, from 83% to 29%, as the pH increased from 3 to 10. The same trend was observed with FeCr-SBA-15, where the degradation efficiency was reduced from 78% to 51%. The maximum degradation of MO was observed at pH = 3. MO has a pKa value of 3.46 [53]. At pH = 3, both the hybrid mixture surface and FeCr-SBA-15 become positively charged because more H^+^ ions are present in the solution, which favors the electrostatic interaction between both surfaces and MO anionic dye. As a result of improved adsorption, a larger quantity of MO dye molecules becomes accessible to the active sites of the photocatalyst. As pH increases, the photocatalytic degradation decreases because the MO dyes are considerably repelled by the predominant negatively charged functional groups present on the surface of FeCr-SBA-15/AC and FeCr-SBA-15. Furthermore, in basic medium (pH > 5), the degradation efficiency was reduced due to the decrease in the oxidation potential of hydroxyl radicals [54]. H_2_O_2_ rapidly degrades into H_2_O and O_2_ at high pH, which reduces its oxidizing capability.

### 2.13. Adsorption Isotherms of AC

The equilibrium isotherm was performed to understand the adsorption mechanism and describe the interaction between MO dyes and AC. In this study, Langmuir and Freundlich isotherm models were used to study the equilibrium data for the adsorption of MO.

The Langmuir model assumes monolayer adsorption onto a homogeneous surface with identical sites without interaction between the adsorbed molecules. However, the Freundlich isotherm suggests that multilayer adsorption takes place on a heterogeneous surface with an irregular dispersion of adsorption energy. The linear form of Langmuir and Freundlich isotherms are represented by Equations (3) and (4), respectively.

(3) $$\frac{C_{e}}{q_{e}}=\frac{1}{q_{m}}C_{e}+\frac{1}{q_{m}.k_{L}}$$

(4) ln⁡qe=1nln⁡Ce+ln KF⁡ 

where *C_e_* represents the equilibrium concentration of MO in solution (mg L^−1^), *q_e_* represents the amount of MO adsorbed at equilibrium time per unit mass of AC (mg g^−1^), *q_max_* is the maximum adsorption capacity (mg g^−1^), *K_L_* is Langmuir constant (L mg^−1^), *K_F_* is related to the adsorption capacity of AC (mg g^−1^) and *n* measures the adsorption intensity.

**Figure 13:**
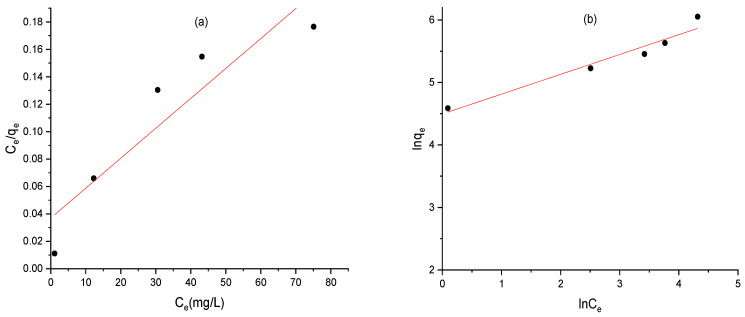
Isotherm linear plots for the adsorption of MO onto AC: (**a**) Langmuir and (**b**) Freundlich.

The linear isotherm plots for MO adsorption onto AC are represented in Figure 13, and the isotherm parameters obtained from the linear fitting are listed in Table 2. The results showed that the correlation coefficient *R*^2^ value of the Freundlich model (0.946) is higher than that of the Langmuir model (0.856). Thus, the isotherm equilibrium data are well represented by the Freundlich isotherm model. These results indicate that the adsorption of MO occur on heterogeneous surfaces with multilayer formation. Additionally, the value of (1/*n*) is 0.317, lies in the range between 0 and 1, which indicates the favorable adsorption of MO onto the heterogeneous surface of AC.

### 2.14. Kinetic Study of MO

To quantitatively investigate the reaction kinetics of MO using the hybrid system and FeCr-SBA-15, the experimental data were fitted to kinetic models. In the present study, three kinetic models—zero, first, and second order—were used to study the degradation kinetics of MO dye using FeCr-SBA-15 and FeCr-SBA-15/AC hybrid mixture (Equations (5)–(7)) [55].

(5) $$C_{t\;}=C_{0}-K_{0}t\;$$

(6) $$\ln\left(\frac{C_{0}}{C_{t\;}}\right)=K_{1}t$$

(7) $$\frac{1}{C_{t}}=\frac{1}{C_{0}}+K_{2}t$$

where *C*_0_, *C_t_* are the initial and final concentrations of MO, respectively; *K*_0_, *K*_1_ and *K*_2_ are the zero, first, and second order rate constant; and *t* is the reaction time (min).

The kinetic parameters for the selected models are listed in Table 3. Comparatively, the correlation coefficient (*R*^2^) value of the first order is greater than zero and second order, which shows that the photocatalytic degradation of MO followed the Langmuir–Hinshelwood pseudo-first-order kinetic model. Notably, the first-order rate constant (*K*_1_) for the hybrid system is 0.0173 min^−1^, which is significantly higher than that of the FeCr-SBA alone at 0.0085 min^−1^. This indicates a more than twofold increase in the rate of MO degradation when AC is incorporated, highlighting the synergistic effect of the adsorbent within the catalytic framework. Pseudo-first-order kinetic model is commonly validated in the literature for similar photocatalytic degradation of MB. For instance, the photodegradation of MB by CdSe nanoparticles is reported to follow a pseudo-first-order kinetic model with a rate constant of 0.038 min^−1^ [56]. Comparable studies using TiO_2_ for MB degradation under similar acidic conditions (pH 5) have demonstrated rate constants of 0.017 min^−1^ [57], further supporting the selection of this model. Moreover, using silver-activated carbon (Ag-AC), MB degradation also adhered to a first-order model with a rate constant of 0.0218 min^−1^ [58].

### 2.15. Possible Degradation Mechanism of MO by Hybrid Mixture

**Figure 14:**
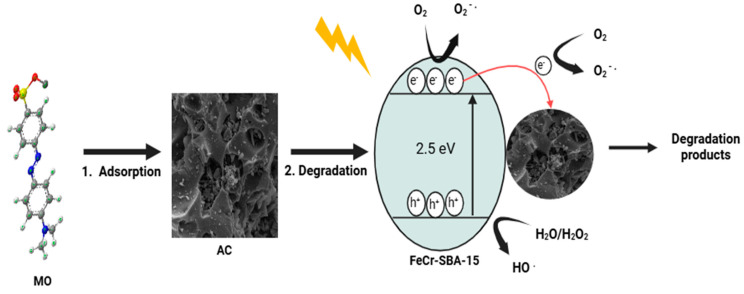
Schematic representation of the synergistic effect of adsorption and photo-Fenton degradation of MO using the hybrid mixture.

The photo-Fenton degradation mechanism of methyl orange (MO) using the hybrid mixture is illustrated in Figure 14. First, MO molecules are adsorbed onto the surface of AC, increasing their local concentration near the FeCr-SBA-15 photocatalyst and facilitating the degradation process. Upon visible light illumination, the FeCr-SBA-15 photocatalyst generates electron (e^−^) in the conduction band (CB) and hole (h^+^) in the valence band (VB). After the light-induced process, the photogenerated electrons are rapidly transferred to AC, which acts as an electron acceptor [59]. This transfer prevents the recombination of electron-hole pairs, thereby enhancing the degradation efficiency [60]. The electrons on the surface of FeCr-SBA-15, along with those transferred to AC, react with adsorbed oxygen (O_2_), leading to the formation of superoxide radicals (O_2_•^−^). Additionally, the holes in the VB of FeCr-SBA-15 interact with water (H_2_O) and hydrogen peroxide (H_2_O_2_) to produce hydroxyl radicals (•OH). The generated radicals (O_2_•^−^ and •OH) attack and break down MO into smaller and less harmful products. Therefore, the synergistic effect of AC and FeCr-SBA-15 enhances the degradation of MO. While FeCr-SBA-15 serves as an efficient photocatalyst, AC enhances MO adsorption, promotes electron transfer, and minimizes electron-hole recombination, ultimately improving the overall degradation efficiency.

### 2.16. Comparison with Other Studies

Table 4 compares the results of our study with previous studies using various photocatalysts for MO degradation. The obtained results show that our prepared hybrid mixture FeCr-SBA-15/activated carbon exhibits significant degradation of MO under visible light irradiation, without changing the pH and using an economical catalyst quantity.

## 3. Materials and Methods

### 3.1. Chemicals

Hydrochloric acid (37% *w*/*w*), polyethylene glycol Pluronic (P123), tetraethyl orthosilicate (TEOS, 99%), hexane, iron nitrate (III) nonahydrate, chromium nitrate (III) nonahydrate, methyl orange, ZnCl_2_, H_2_O_2_ (35% *w*/*v*), and NaOH were purchased from Sigma-Aldrich (Beirut, Lebanon).

### 3.2. Synthesis of SBA-15 Support

The SBA-15 samples were synthesized by the sol-gel method under acidic conditions using P_123_ as the template and TEOS as a silica source. The mesoporous silica SBA-15 was synthesized by adopting the procedure reported by Tabaja et al. [28]. In this method, distilled water was acidified with HCl (37%), and 16.7 g of P123 was then dissolved in the obtained acidic solution. The thermostat connected to the reactor was set to 35 °C, ensuring continuous stirring throughout the process. After the complete dissociation of P123, a homogenous solution was formed. Then, 42 mL of TEOS was added dropwise to the solution. The stirring was stopped immediately after TEOS addition, and the temperature was kept at 35 °C for 24 h. The solution was then hydrothermally treated at 130 °C for 33 h in a 500 mL autoclave. To remove the organic template, the sample was calcined at 550 °C for 5 h using a heating rate of 2 °C min^−1^. Finally, the product was calcined for 6 h at 500 °C with a heating rate of 2 °C min^−1^. The calcined SBA-15 samples were recovered and stored away from moisture to be further used in the doping process.

### 3.3. Synthesis of Chromium Ferrite Supported on the Mesoporous SBA-15

Chromium ferrite nanoparticles were synthesized from calcined SBA-15 using a double solvent replication technique. For this synthesis, 0.88 g of freshly calcined SBA-15 was immersed in 35 mL of cyclohexane in a large alumina mortar and mixed thoroughly with a pestle. Then, 0.85 mL of an aqueous Fe(NO_3_)_3_·9H_2_O solution (1.7 M) was added drowise, followed by the addition of 0.85 mL of an aqueous Cr(NO_3_)_3_·9H_2_O solution (0.91 M), achieving final concentrations of 8 wt.% Fe and 4 wt.% Cr in the product. This mixture was homogenized to ensure the precursor salts penetrated the SBA-15 pores via repulsive interactions with the organic solvent. The cyclohexane was subsequently decanted, and the dried catalyst was then calcined in a muffle furnace, heated at 2 °C min^−1^ up to 700 °C for 5 h, and then allowed to cool to room temperature. The final product, named FeCr-SBA-15, utilized quenching to create lattice gaps and prevent large crystal formation.

### 3.4. Preparation of Activated Carbon

The AC was prepared using the process reported by Hamieh et al. [68]. The collected olive mill wastes were first washed thoroughly with distilled water and then sun-dried. After drying, the wastes were ground into fine particles using an IKA Werke grinding machine. These fine particles were then combined with a ZnCl_2_ solution at an impregnation ratio of 2:1 (mass ZnCl_2_ to mass OMSW) and allowed to sit for 24 h at room temperature. Subsequently, the mixture was dried in an oven at 110 °C for 24 h. The impregnated material was then placed in a closed crucible and exposed to heat treatment in a controllable electric muffle furnace at 500 °C for 2 h with a heating rate of 5 °C per minute. Once the heating process was complete, the activated carbon (AC) produced was allowed to cool, then washed with 0.1 M HCl solution and deionized water to remove any remaining chloride and mineral impurities. Finally, the AC was dried again in an oven at 110 °C for 24 h and stored for future use.

### 3.5. Preparation of Hybrid Mixture (FeCr-SBA/AC)

The hybrid mixture (FeCr-SBA-15/AC) was prepared using a set of different FeCr-SBA-15/AC powder mass ratios (90/10, 70/30, 50/50, and 10/90). A known mass of FeCr-SBA-15 and AC with different mass ratios as mentioned were placed inside a mortar and physically blended using a pestle to obtain fine powder.

### 3.6. Technique Characterization

The crystal structure of the catalysts was characterized by XRD using Bruker D8 (Karlsruhe, Germany) advance diffractometer with a Cu Kα beam source (λ = 1.541 Å) in the 2*θ* range of 0–60° at 40 kV and 40 mA. The morphologies of catalysts were assessed using scanning electron microscope (SEM, FEI Quanta FEG 250, Hillsboro, OR, USA) with an accelerating voltage of 20 kV and transmission electron microscope (TEM, JEOL, JEM-2100F, Akishima, Tokyo, Japan) at an accelerating voltage of 200 kV. The pore structure of the prepared catalysts was analyzed through measurement of the N_2_ adsorption–desorption isotherm using a Micromeritics 3Flex Surface Analyzer (Norcross, GA, USA) at 77 K. Before analysis, all samples were subjected to vacuum degassing at 200 °C overnight. The specific surface area (*S_BET_*) was estimated by the BET equation within a relative pressure range of 0.01–0.1. Pore size was determined by the Barrett–Joyner–Halenda (BJH) method applied to the desorption branch of the isotherm and the t-plot method was used to determine micropore volume. A Perkin Elmer Lambda 900 spectrophotometer (Shelton, CT, USA) was employed to obtain the diffuse reflectance spectra, and MgO was used as a reference. The spectra obtained were transformed using the Kubelka–Munk model to determine the band gap. FTIR was recorded using Nicolet Summit-LITE FTIR spectrometer (Madison, WI, USA) over a spectral range of 400–4000 cm^−1^ at a resolution of 4 cm^−1^.

### 3.7. Photo-Fenton Experiments

**Figure 15:**
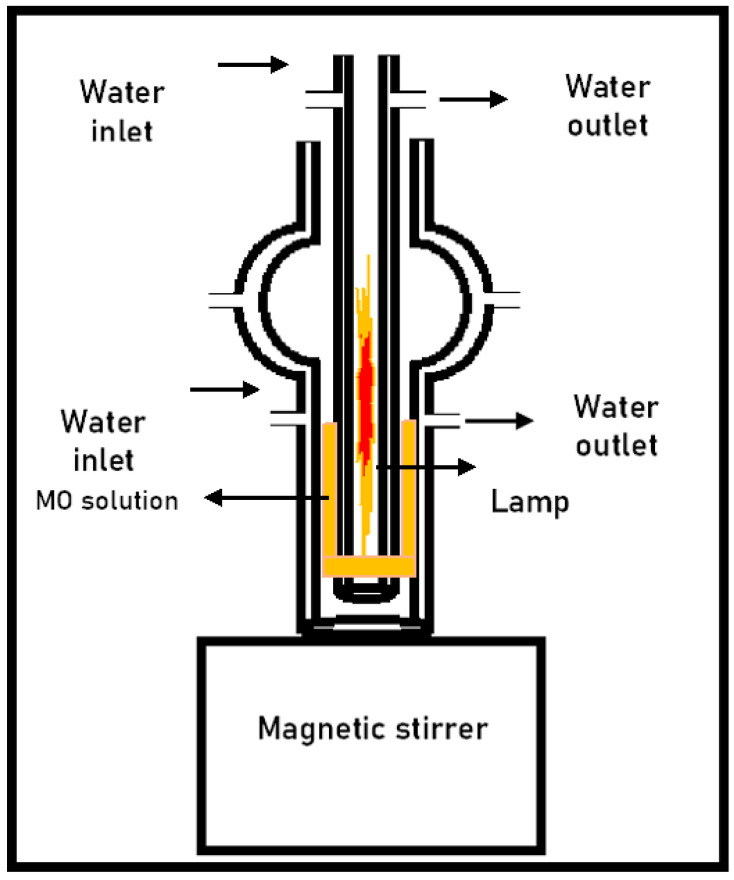
Schematic of photocatalytic reactor.

The degradation of methyl orange via photo-Fenton was conducted under visible light using a halogen RJH-TD Radium lamp (100 W/230 V/c/E27) (Wipperfürth, North Rhine-Westphalia, Germany) in a reactor with a double cooling system (Figure 15). For the degradation process of MO pollutant, 75 mg of FeCr-SBA-15/AC was dispersed inside the reactor containing 99.5 mL of aqueous MO solution with a concentration of 20 ppm and the water source was opened. After a 30 min period to allow for the adsorption equilibrium of MO, 0.5 mL of H_2_O_2_ was introduced into the reactor, marking the start of the reaction. Samples were collected from the beginning to 240 min at various intervals. The samples were then analyzed after filtration through 0.45 µm filter syringe. The filtered dye solutions were analyzed to determine the residue MO concentration by recording the absorbance at 464 nm using a UV-visible spectrophotometer (Madison, WI, USA). The degradation efficiency was calculated using Equation (8).

(8) $$Degradation\;efficiency\left(\%\right)=\frac{{(C}_{0-}C_{t})}{C_{0}}\times 100\;$$

where *C*_0_ is the initial concentration of dye (mg L^−1^) and *C_t_* is the concentration of dye at time interval (mg L^−1^).

## 4. Conclusions

In this study, an effective hybrid mixture FeCr-SBA-15/activated carbon was utilized for degradation of MO from an aqueous solution with high optical density under visible light irradiation. FeCr-SBA-15 was synthesized by the double solvent method. UV-Vis spectrophotometry, coupled with the Kubelka–Munk method and Tauc plot analysis, revealed band-gap value of 2.5 eV for the synthesized semiconductors. This observation substantiated the potential applicability of the synthesized photocatalyst in visible light photocatalysis. Olive mill solid wastes which may cause environmental pollution if not disposed of properly, were converted to AC by chemical activation using ZnCl_2_. The hybrid mixture was prepared by physical mixing. The hybrid mixture exhibited excellent photo-Fenton degradation of MO under visible light using a low concentration of H_2_O_2_. Almost 97% of MO was degraded within 180 min using the hybrid mixture (90:10) under the following experimental conditions (hybrid mixture dosage = 0.75 g L^−1^, 0.5 mL H_2_O_2_, [MO] = 20 ppm, and pH = 5.46). The presence of AC in the mixture provides a synergistic effect and can suppress the electron-hole recombination. The degradation of MO was influenced by several parameters such as reaction time, hybrid mixture dosage, initial dye concentration, solution pH, and amount of H_2_O_2_. The kinetic experiments showed that the photo-Fenton degradation using the hybrid mixture followed the first-order kinetic model expressed by Langmuir–Hinshelwood. In future studies, we plan to evaluate the activity of the hybrid mixture under natural sunlight to minimize energy use during the photo-Fenton degradation of MO. Should these tests confirm its efficacy, our hybrid mixture may be deemed a viable photocatalyst for treating organic pollutants with high optical density. Furthermore, we intend to investigate the capacity of this catalyst to break down other persistent organic pollutants, such as antibiotics, which are particularly challenging to eliminate from contaminated water sources. Such research could substantially expand the utility of our catalyst in various environmental remediation applications.

## Figures and Tables

**Table 1:** Summary of textural properties measured by N_2_ sorption at 77 K.

Sample	*S_BET_* (m^2^ g^−1^)	*V_p_^a^* (cm^3^ g^−1^)	*D_pore_ ^b^* (nm)	*V_µp_^c^* (cm^3^ g^−1^)
SBA-15	522	1.48	8.7	0.013
FeCr-SBA-15	372	0.65	6.8	0.006
AC	1148	0.6	3.2	0.055

*^a^* porous volume, a single point determined at P/P_0_ 0.98. *^b^* BJH pores size distribution calculated from desorption branch. *^c^* micropore volume determined from t-plot.

**Table 2:** Isotherm parameters of Langmuir and Freundlich models.

Isotherm Models	Parameters	Values
Langmuir	*q_m_*	458.71
	*K_L_*	0.059
	*R* ^2^	0.856
Freundlich	*n*	3.15
	*K_F_*	89.73
	*R* ^2^	0.946

**Table 3:** Kinetic parameters for the degradation of MO using hybrid mixture FeCr-SBA-15/AC.

Kinetic Models	Parameters	FeCr-SBA/AC	FeCr-SBA
Zero order	*K*₀	0.0439	0.061
	*R* ^2^	0.8632	0.9356
First order	*K*₁	0.0173	0.0085
	*R* ^2^	0.9912	0.9982
Second order	*K*_2_	0.0206	0.0016
	*R* ^2^	0.8295	0.8896

**Table 4:** Comparison of different photocatalysts for the degradation of MO.

Photocatalyst	Catalyst Concentration (g L^−1^)	MO Concentration (ppm)	pH	Light Source	MO Degradation (%)	Reaction Time (min)	Reference
HPW-Fe-Bent	0.75	10	-	UV light (40 W)	78.1	60	[61]
α-Fe_2_O_3_	1	20	3	UV lamp (15 W)	82.17	100	[62]
MoS_2_/Co_3_O_4_	0.2	20	-	Xe lamp (350 W)	95.6	170	[63]
NiFe_2_O_4_/SiO_2_/NiO	0.5	10	4	UV light (40 W)	95.76	120	[64]
g-C_3_N_4_/biochar	0.75	10	3.48	LED (20 W)	96.63	30	[65]
High-silica SAPO-5	1.4	10	-	Hg lamp (36 W)	82.8	240	[66]
TiO_2_/ZSM-5	2	20	7.5	solar simulator (100 mW cm^−2^)	99	180	[67]
FeCr-SBA-15/activated carbon	0.75	20	6.47	Halogen Lamp (100 W)	97	180	This study

## Data Availability

There is no additional data.
